# Characterization of Forearm Muscle Activation in Duchenne Muscular Dystrophy via High-Density Electromyography: A Case Study on the Implications for Myoelectric Control

**DOI:** 10.3389/fneur.2020.00231

**Published:** 2020-04-15

**Authors:** Kostas Nizamis, Noortje H. M. Rijken, Robbert van Middelaar, João Neto, Bart F. J. M. Koopman, Massimo Sartori

**Affiliations:** ^1^Department of Biomechanical Engineering, Technical Medical Centre, University of Twente, Enschede, Netherlands; ^2^Faculty Physical Activity and Health, Saxion University of Applied Sciences, Enschede, Netherlands; ^3^Faculty of Sciences, University of Lisbon, Lisbon, Portugal

**Keywords:** Duchenne muscular dystrophy, forearm, hand, high-density surface electromyography, motor control, myocontrol, principal component analysis (PCA), wrist

## Abstract

Duchenne muscular dystrophy (DMD) is a genetic disorder that results in progressive muscular degeneration. Although medical advances increased their life expectancy, DMD individuals are still highly dependent on caregivers. Hand/wrist function is central for providing independence, and robotic exoskeletons are good candidates for effectively compensating for deteriorating functionality. Robotic hand exoskeletons require the accurate decoding of motor intention typically via surface electromyography (sEMG). Traditional low-density sEMG was used in the past to explore the muscular activations of individuals with DMD; however, it cannot provide high spatial resolution. This study characterized, for the first time, the forearm high-density (HD) electromyograms of three individuals with DMD while performing seven hand/wrist-related tasks and compared them to eight healthy individuals (all data available online). We looked into the spatial distribution of HD-sEMG patterns by using principal component analysis (PCA) and also assessed the repeatability and the amplitude distributions of muscle activity. Additionally, we used a machine learning approach to assess DMD individuals' potentials for myocontrol. Our analysis showed that although participants with DMD were able to repeat similar HD-sEMG patterns across gestures (similarly to healthy participants), a fewer number of electrodes was activated during their gestures compared to the healthy participants. Additionally, participants with DMD activated their muscles close to maximal contraction level (0.63 ± 0.23), whereas healthy participants had lower normalized activations (0.26 ± 0.2). Lastly, participants with DMD showed on average fewer PCs (3), explaining 90% of the complete gesture space than the healthy (5). However, the ability of the DMD participants to produce repeatable HD-sEMG patterns was unexpectedly comparable to that of healthy participants, and the same holds true for their offline myocontrol performance, disproving our hypothesis and suggesting a clear potential for the myocontrol of wearable exoskeletons. Our findings present evidence for the first time on how DMD leads to progressive alterations in hand/wrist motor control in DMD individuals compared to healthy. The better understanding of these alterations can lead to further developments for the intuitive and robust myoelectric control of active hand exoskeletons for individuals with DMD.

## Introduction

Duchenne muscular dystrophy (DMD) is an X chromosome-linked recessive neuromuscular disease (1). The absence of dystrophin causes progressive weakness of skeletal, respiratory, and cardiac muscles and leads to severe physical disability and shortened life expectancy (2). Improved care standards and the recent introduction of assisted ventilation, in the later stages of the disease, contributed to the increase of their life span (3). This has led to increasing numbers of adults with DMD (4) who experience low quality of life and external aid dependency (5, 6).

In DMD individuals, the support of the upper extremity is central for ensuring daily life independence (7). Wearable devices such as hand/wrist exoskeletons can provide a functional solution by assisting individuals with DMD in performing activities of daily living (ADL) (7). However, dynamic active hand support currently remains a challenge (4), with passive hand orthoses (8) still representing the main clinical approach. Bushby et al. (9, 10) suggested that the treatment of individuals with DMD should become more multidisciplinary as well as promote further the use of technology. However, the effective use of active orthoses requires the accurate decoding of motor intention, which represents an important yet not well-addressed challenge (11).

The clinical golden standard for non-invasive motor intention decoding (12), control of robotic devices (13), and characterization of muscle activity (14) is low-density surface electromyography (sEMG). The most common approach involves bipolar sEMG, where muscle activation is measured with the placement of two closely placed electrodes above the muscle belly (15, 16). sEMG is currently biased by superposition of electrical potentials that compromise signal amplitude estimation, the need for identifying optimal electrode placement, skin–electrode impedance, power line interference, and physiological properties (intermuscular fat, skin humidity, etc.) (15). Despite the fact that sEMG is broadly used in amputee research (14, 17–19) to characterize forearm activity, in degenerative disorders such as DMD, there is a lack of understanding on how these individuals activate their forearm muscles to achieve functionally relevant tasks. A possible way to address this challenge is the use of high-density sEMG (HD-sEMG).

HD-sEMG is a non-invasive technique that collects high-resolution myoelectric signals from tens of monopolar electrodes, i.e., >60 electrodes simultaneously (20). With respect to conventional low-density approaches, HD-sEMG enables determining how large muscles, such as those in the human forearm, activate not only in the temporal domain but also in the spatial domain (14). This information can be used to create heatmaps encoding the spatial distribution of HD-sEMG amplitudes during different hand/wrist-related tasks (19). Such heatmaps can capture distinct HD-sEMG patterns associated to specific tasks, plus variations in amplitude, and repeatability over time. This is central for taking into account the manifestation of inhomogeneities in the control of the muscular fibers, something crucial to understand in pathological muscle activation (21). Moreover, this can be used to explore myocontrol in pathological populations when combined with currently used machine learning classification techniques (22). Currently, HD-sEMG is performed with a large number of cables and is biased by heavy and sizable amplifiers which limit its use in dynamic situations, such as the control of wearable exoskeletons (23).

HD-sEMG spatiotemporal analysis and pattern recognition were never applied to DMD individuals. The use of HD-sEMG can give insights in dimensionality and spatiotemporal similarity between healthy and DMD participant and additionally open a window to study hand/wrist motor control in DMD via a number of analyses and understand the hierarchical motor control in DMD and differences with respect to healthy people. Repeatability, spatial distribution, and distinguishability of HD-sEMG patterns together with HD-sEMG classification performance are important requirements for understanding the altered DMD motor control and use our findings in the context of robotic exoskeleton applications.

In this paper, we characterize HD-sEMGs of three individuals with DMD during seven hand/wrist-related tasks and compare with a baseline of eight healthy participants. This work is motivated by the near absence of a systematic and detailed spatiotemporal characterization of forearm muscle activations in individuals with DMD. First, we create HD-sEMG heatmaps and analyze them with principal component analysis (PCA) to identify the number of orthogonal muscle activation spatiotemporal patterns. Second, we characterize the ability of DMD individuals to produce repeatable and spatiotemporally distinguishable HD-sEMG patterns across tasks, as well as their amplitude distribution. Third, we employ pattern recognition to quantify the potential of each DMD individual to perform activities as those required for the control of assistive robotic exoskeletons. We hypothesize that participants with DMD will show lower activations, they will perform less repeatable patterns and show differences in dimensionality because DMD's central nervous system (CNS) acts on an impaired musculoskeletal apparatus, which may in turn lead to CNS adaptations. Finally, we hypothesize that myocontrol performance will be lower in DMD compared to the healthy participants.

## Materials and Methods

### Participants

The experiment was carried out by seven healthy female and one male adults (age: 21.4 ± 1.2 years, forearm length: 24.8 ± 1.8 cm, forearm circumference at 20% of length: 25.9 ± 1.8 cm), without any hand-related impairment, and three male adults with DMD (age: 22.3 ± 2.5 years, forearm length: 24.2 ± 2.9 cm, forearm circumference at 20% of length: 25.5 ± 3.9 cm).

The DMD participants had different levels of hand function. Participant one (DP1, 20 years old) was able to use his hands functionally, and no contractures relevant to hand/wrist movement were observed. Participant two (DP2, 22 years old) was able to functionally use his hand but experienced a decrease in strength and minimal contractures relevant to hand/wrist movement. Participant three (DP3, 25 years old) was not able to use his hands at all and was affected by immediate onset of fatigue during its use. Extensive contractures relevant to finger movement were observed, and only minimal movement of the fingers was possible (see Supplementary Video). All participants were able to perform the experimental protocol.

The Medical Ethics Committee of Twente approved the study design, the experimental protocol, and the procedures (Protocol number: NL59061.044.16). The study was conducted according to the ethical standards given in the Declaration of Helsinki in 1975, as revised in 2008.

### Experimental Setup and Signal Acquisition

**Figure 1:**
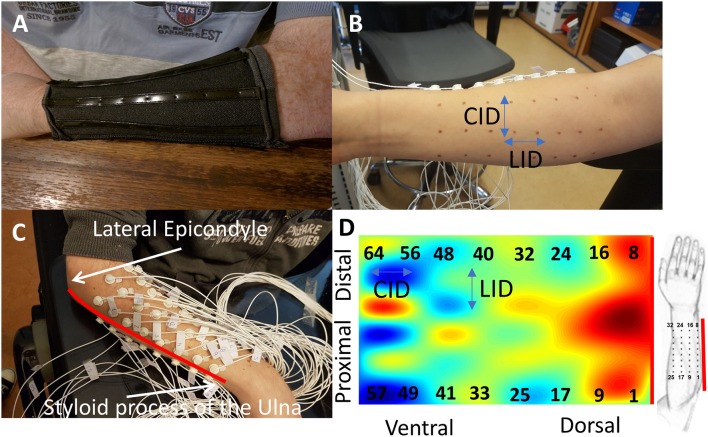
The figure shows the process of the electrode placement. **(A)** The flexible custom-made sleeve that was used for marking the skin of the participant. The sleeve is flexible only around the circumferal direction and stiff along the longitudinal direction of the arm. **(B)** The marked skin of the participant. The longitudinal inter-electrode distance (LID) is fixed at 2 cm (L), while the circumferal inter-electrode distance (CID) depends on the forearm width of each participant. **(C)** The participant with all the 64 electrodes placed. The imaginary line (red line) that connects the lateral epicondyle and the styloid process of the ulna was used as the border between the dorsal and ventral side of the forearm. The placement of the electrodes starts right above this line, with electrode number one placed proximally (at 20% of forearm length from the elbow) and eight distally. The rest of the electrode rows are placed counterclockwise as someone is looking at his right arm. **(D)** This way, electrodes 1–32 were placed over the dorsal side (see sketch) and 33–64 over the ventral side of the forearm. The center of gravity (COG) is also shown for this gesture.

The experimental setup (Figure 1) included several components, and it was designed to record HD-sEMG signals from the forearm in a repeatable and systematic way. Muscular activity was measured with a 128-channel amplification system (REFA 128 model, TMS International, Oldenzaal, The Netherlands). We used 64 monopolar electrodes around the forearm to acquire the raw sEMG signals. The signals were recorded with a decimal gain of 26.55 before the analog-to-digital converter (ADC); however, this gain factor is compensated by the acquisition software (Polybench, TMS International, Oldenzaal, The Netherlands), after the ADC. Additionally, REFA includes a first-order analog low-pass filter placed before the ADC with a −3 db point at 6.8 kHz. The 6.8-kHz low pass helps to make the REFA immune to high-frequency electromagnetic interference such as mobile phone networks. The analog signals were sampled with a frequency of 2,048 Hz and digitally converted with a 24-bit conversion (a resolution of 0.018 μV per bit, 300 mV dynamic range). The ADC of the device has an anti-aliasing digital low-pass filter with a cutoff frequency of 0.2 ^*^ sample frequency. This filter inside the ADC is used to convert the 1-bit signal with a high frequency into a 24-bit signal with a lower frequency. The acquisition software was executed in a host laptop (Lenovo Thinkpad T490, Lenovo, Beijing, China) with a Windows 10 operating system (Microsoft Corporation, Washington, USA). A computer screen was used to provide visual feedback of the task to the participants.

Electrode placement and configuration were based on previous work (19) that normalized the electrode locations to each participant's arm circumference in order to account for different forearm thicknesses (Table 1). The inter-electrode distance in the longitudinal direction of the forearm was kept constant at 2 cm for covering the entire forearm (24).

**Table 1:** Participant information.

**Participant**	**Dominant arm**	**Forearm length (20%) (cm)**	**LID (cm)**	**At 20% of forearm length from the elbow**	
				**Forearm circumference (cm)**	**CID (cm)**
HP1	R	26 (5.2)	2	27	3.38
HP2	R	23 (4.6)	2	24	3
HP3	R	28 (5.6)	2	26	3.25
HP4	R	26 (5.2)	2	27	3.38
HP5	R	22.5 (4.5)	2	23.5	2.94
HP6	R	24 (4.8)	2	25	3.13
HP7	R	24 (4.8)	2	26	3.25
HP8	R	25 (5)	2	29	3.63
DP1	L	23 (4.6)	2	27.5	3.4
DP2	R	27.5 (5.5)	2	28	3.5
DP3	R	22 (4.4)	2	21	2.63

*HP denotes the healthy participants and DP the participants with Duchenne muscular dystrophy (DMD). LID and CID denote the longitudinal and circumferal inter-electrode distance, respectively*.

First, we cleaned the skin of the dominant forearm of the participant with alcohol. Then, we measured the forearm length from the lateral epicondyle until the styloid process of the ulna and the forearm circumference at 20% of the forearm length from the elbow (Figure 1). The participant had to wear a perforated sleeve (Figure 1) with equally placed holes and elastic only along the circumferal direction to ensure that the electrode placement was standardized for all participants. We used a non-permanent marker to mark the skin of the participant (Figure 1) and then visually inspect the markings before applying the electrodes.

Conductive gel was applied to each of the 64 electrodes with a syringe, and they were subsequently attached to the forearm. The first row of electrodes was placed above the imaginary line between the lateral epicondyle and the styloid process of the ulna and the last row below in such a way that the line lay in the middle between the two rows of electrodes (Figure 1). The first electrode was attached proximally starting at the 20% of the forearm length from the elbow. Electrodes were placed from proximal to distal and in counterclockwise direction (from the perspective of a right-handed participant). This way, electrodes 1–32 were placed over the dorsal side (mostly extensor muscles) and 33–64 over the ventral side (mostly flexor muscles) of the forearm. The reference electrode was placed at the distal end of the forearm, over the head of the ulna.

**Figure 2:**
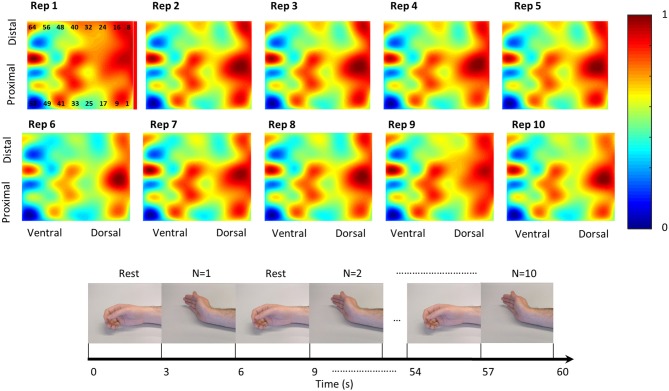
The 10 repetitions of third Duchenne muscular dystrophy (DMD) participant (DP3) for wrist extension that were used to acquire the average normalized map. The lower part shows an example of the protocol followed to record the data. In this example, the participant was instructed to extend his wrist for 3 s and then rest for 3 s. This was repeated 10 times. The same procedure was followed for all the seven gestures.

Participants performed seven different gestures involving hand and wrist motions (Figure 2). The chosen gestures included: hand open/close, thumb flexion/extension, wrist flexion/extension, and index extension. These were chosen as they are involved in the most frequent ADL (25). First, each participant was instructed to perform all gestures without constraints (dynamic) with maximal voluntary effort in a single recording. This way, we recorded the maximum voluntary contraction (MVC) for every electrode across all gestures. For every gesture, 10 repetitions of 3 s contractions were performed, together with 10 repetitions of 3-s resting periods between the contractions (Figure 2). The participants were instructed to perform all movements in a comfortable fashion in order to avoid forceful contractions that may elicit co-contractions of agonist–antagonist muscle groups.

The timing of the gestures was dictated with the use of visual feedback. The visual feedback illustrated via photographs of human hands which gesture had to be performed. The sequence of images served to instruct the participant as a metronome when to perform the gesture (image of gesture appearing for 3 s) and when to relax (image of relaxed hand appearing for 3 s). Additionally, the measurements were performed in the morning in order to avoid effects of the end-of-the-day fatigue, especially for the participants with DMD. Furthermore, the participants had short breaks between gestures in order to rest.

### Signal Processing and Analysis

All signal processing and data analyses were performed in Matlab 2018b software (The MathWorks Inc., USA). The raw sEMG signals were processed offline in order to compute the envelopes for each of the 64 electrodes per gesture and per participant. First, the raw data were filtered with a band-pass filter (fourth-order Butterworth, 20–450 Hz). Additionally, a second-order digital infinite impulse response notch filter (cutoff frequency of 50 Hz, Q factor of 50) was used to remove the power line noise (50 Hz for the EU). Despite its main limitation (signal distortion around the attenuated frequency), notch filtering is the mainstream technique for powerline signal removal (26), and a narrow bandwidth with a high Q factor can already address this (27). For highly powerline-contaminated signals, spectral interpolation may be more appropriate (27). The signals were subsequently rectified and filtered with a low-pass filter (third-order Butterworth, 2 Hz). Our choice for the cutoff frequency was motivated by the low-frequency dynamic tasks involved in this study (28) and our previous study on real-time sEMG control of a hand exoskeleton (29). The resulting envelopes were visually inspected segmented, according to the acquisition protocol, to 10 contractions and periods (each lasting approximately 3 s) and normalized. A threshold was selected to define the onset of the activity, and the next 3 s after the onset were chosen as a contraction period. The threshold was defined as the time that the signal exceeded 10 standard deviations of the baseline (non-contraction) activity similar to Di Fabio (30), and the final segmentation was additionally assessed visually. The maximum value of the envelope of each electrode across the complete dataset was used as a normalization value for each electrode. This value was acquired using a moving average window of 1 s in order to account for signal artifacts. Signal quality was visually assessed both in the time and frequency domains, and faulty channels were replaced by linear interpolation of their surrounding neighboring channels (8-neighborhood) (14). Different local conditions were applied to faulty electrodes placed in the longitudinal extremes (<8 neighboring channels).

**Figure 3:**
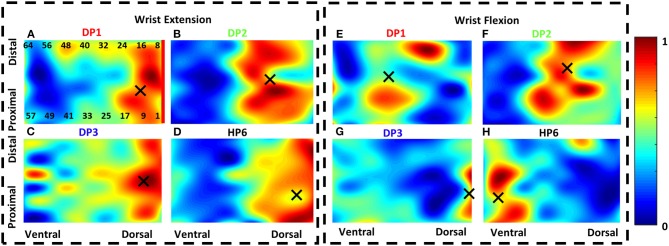
The heatmaps of two representative gestures for the three participants with Duchenne muscular dystrophy (DMD) and one healthy participant. **(A–D)** show wrist extension heatmaps for DP1 **(A)**, DP2 **(B)**, DP3 **(C)**, and HP6 **(D)**. **(E–G)** show wrist flexion heatmaps for DP1 **(E)**, DP2 **(F)**, DP3 **(G)**, and HP6 **(H)**. Regarding wrist extension, all participants exhibit similar activation patterns. However, for wrist flexion, there is higher variability in the activation patterns within participants. X marks show the center of gravity (COG) for each heatmap. Only the activations that are higher than 80% are used to calculate the COG.

Every 3-s contraction was further segmented in 1-s segments by keeping only the middle second of the contraction (steady-state phase) and discarding the transient phase (31). For every electrode, the average of this 1-s contraction was calculated and used to construct 10 heatmaps per gesture (Figure 2). For the visual inspection of the forearm activity per gesture, we constructed activity heatmaps by averaging the 10 repetition heatmaps (Figure 3).

We analyzed the data to assess HD-sEMG pattern repeatability, peaks, and dimensionality, as well as individuals' potential to generate activation patterns suitable for myocontrol applications for both healthy and DMD participants. The raw data used for this analysis are available online (32). All signal processing and data analyses were performed in Matlab 2018b software (The Mathworks Inc., USA). In the remainder of this section, we describe a set of analyses aimed at investigating differences between DMD and healthy participants at the level of motor control properties (*Motor Control Properties* section) and myocontrol performance (*Myocontrol Performance* section).

### Motor Control Properties

#### Activation Pattern Repeatability Tests

**Figure 4:**
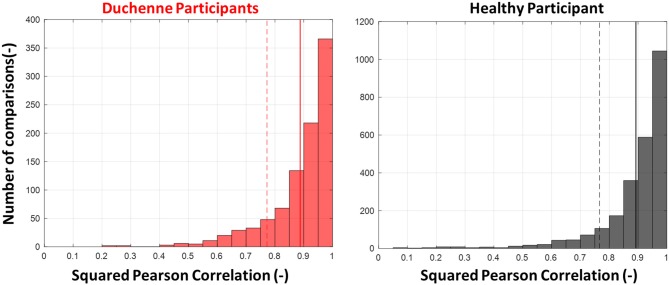
The histogram of squared Pearson correlation between the 10 repetitions for all gestures and for all participants. High correlation shows similarity between the repetitions and thus high repeatability. Both healthy and Duchenne muscular dystrophy (DMD) participants achieved similarly high repeatability on the tasks. The full vertical lines represent the mean and the dashed the standard deviation. The number of unique comparisons between 10 repetitions is 45 multiplied by the seven gestures makes 315 unique comparisons per participant. That explains the total of 2,520 events in the healthy histogram compared to the 945 in the DMD.

The degree of repeatability across repetitions per participant was calculated using squared Pearson correlation. Each heatmap (8 × 8) was reshaped into a vector (1 × 64) before the calculation of the squared Pearson correlation (33). The coefficient was extracted among the 10 repetitions per gesture and per participant. For every gesture, this resulted in 45 unique comparisons between the 10 repetitions and thus 45 coefficients per gesture (Figure 4).

#### Spatiotemporal Activation Pattern Tests

**Figure 5:**
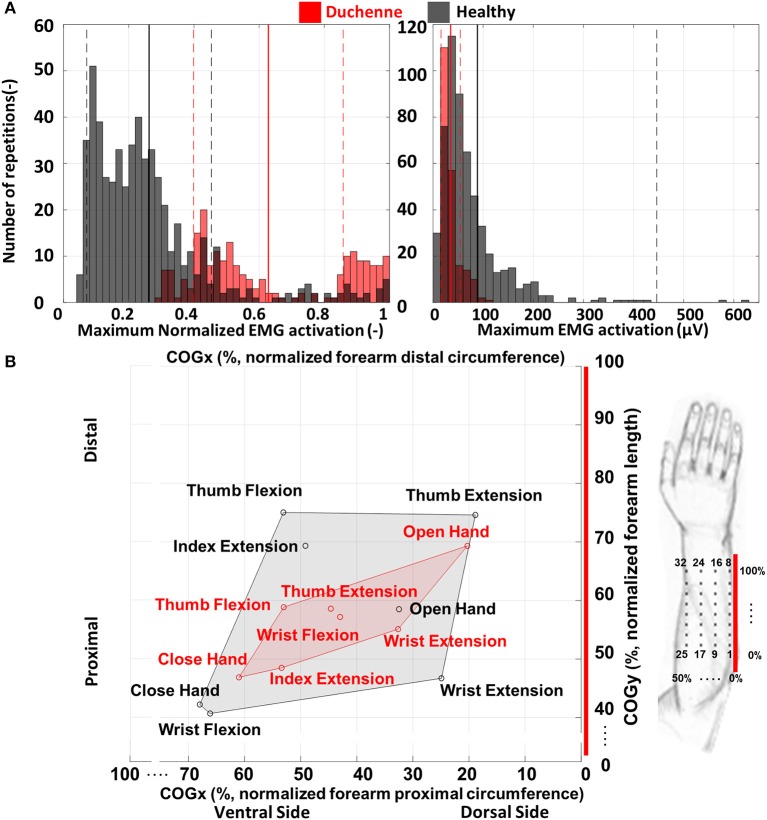
**(A)** The maximum normalized (left) and absolute (right) activation for each of the 10 repetitions of each gesture for all participants. Healthy participants generally performed the tasks with low levels of maximum normalized activation, while participants with Duchenne muscular dystrophy (DMD) showed higher levels of maximum normalized activation during the tasks. However, the maximum absolute activations were higher for the healthy participants. The full vertical lines represent the mean and the dashed the standard deviation. **(B)** The average center of gravity (COG) for the seven gestures for the healthy participants (black) and the participants with DMD (red). Healthy participants (gray shaded area) show on average a broader spatial distribution of the seven gestures than the participants with DMD (red shaded area). The red line represents the imaginary line that connects the lateral epicondyle and the styloid process of the ulna and was used as the border between the dorsal and ventral side of the forearm (see also Figure 1). The COG coordinates are normalized over the forearm circumference (COGx) and length (COGy).

The temporal distribution of activations between healthy and DMD was calculated via normalized and absolute activations per repetition of each gesture (Figure 5A). A normalization factor was calculated across all gestures and repetitions. For each gesture, the maximum absolute and normalized value of the 64-electrode heatmap were calculated for every participant and each repetition and plotted.

Figure 5B shows the average spatial distribution of the healthy and DMD participants. The spatial distribution of the sEMG potentials over the 8 × 8 normalized heatmap was calculated using the center of gravity (COG) by calculating the dorsal–ventral and the proximal–distal position of it as proposed by Elswijk et al. (34). The COG was calculated over electrodes presenting activations equal or larger than 80% of the maximal value of the heatmap (Figure 3). This way, only clusters of electrodes with a high peak amplitude were considered for the calculation of the COG in order to focus on the most relevant area of activation for each gesture.

#### Activation Pattern Dimensionality Tests

**Figure 6:**
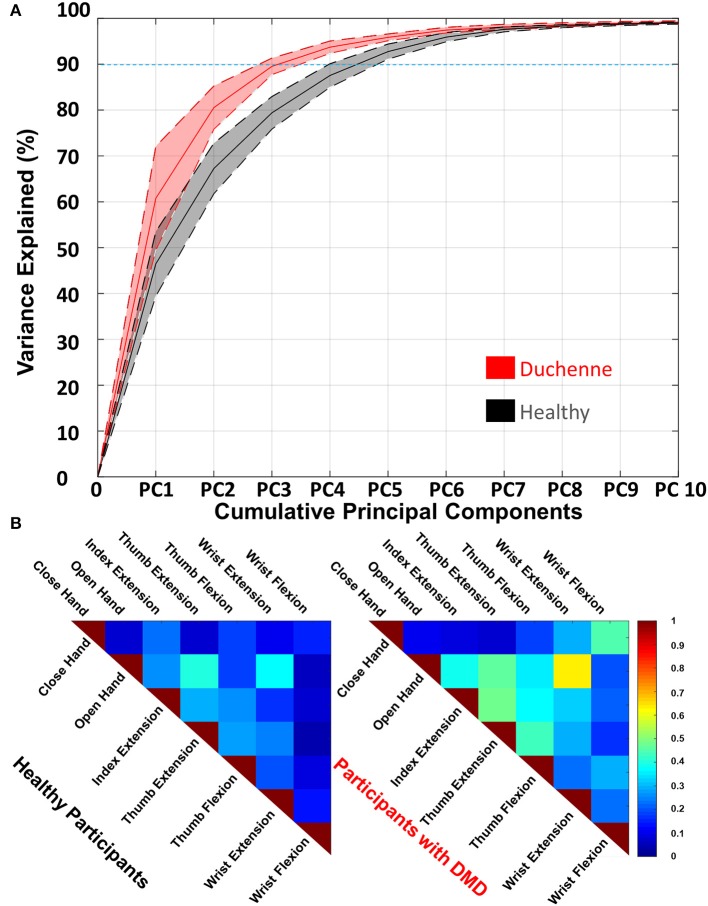
**(A)** The percentage of variance explained as a function of the number of cumulative principal components (PCs). More than 90% of the variance (blue dashed line) of the data of the participants with Duchenne muscular dystrophy (DMD) is explained by three PCs, while for the healthy by five. The full lines represent the mean and the dashed the standard deviation. For clarity, we include only up to 10 of the 63 components, as those explain more than 99% of the variance explained. **(B)** The averaged squared Pearson correlation between the seven gestures of both groups of participants in the form of a similarity matrix. High correlation shows similarity between the gestures. Both healthy and DMD participants show correlated gestures; however, this phenomenon is more prominent in the DMD participants. A high value shows high correlation where one is the maximum (diagonal). The number of unique comparisons between the seven gestures is 21 per participant.

The 10 heatmaps, one per gesture repetition, were used to construct one single average heatmap per gesture per participant (Figure 3) that was used for the motor control analysis. We quantified differences in dimensionality of orthogonal and uncorrelated sEMG patterns between the healthy and DMD participants via a PCA (35) to the gesture-specific heatmaps per participant. For every participant, we performed a PCA to the concatenation (64 × 70) of the sEMG heatmaps of all gestures and repetitions per participant [64 electrodes × (7 gestures × 10 repetitions)]. The number of PCs needed to reconstruct the original seven gesture heatmaps was identified per participant by means of the variance explained (VE), and it was taken as the number of PCs that summed together explained more than 90% of the total variance. This number was used to explore the repertoire of orthogonal and uncorrelated sEMG patterns produced by the two groups of participants (Figure 6A).

Additionally, we calculated the squared Pearson correlation between all the gestures per participant (the same way as we did for the repeatability, *Activation Pattern Repeatability Tests* section). The coefficient was extracted from the average normalized heatmap of the 10 repetitions per gesture and per participant. For every participant, this resulted in 21 unique comparisons between the seven gestures and thus 21 coefficients per participant. We averaged the correlation values of the healthy participants and the participants with DMD separately to identify which gestures are mostly correlated per population, and we presented this in the form of a similarity matrix (Figure 6B).

### Myocontrol Performance

We explored participants' gesture recognition performance via an offline pattern recognition algorithm applied to the band-pass filtered data (fourth-order Butterworth, 20–450 Hz) of each participant. We used a linear discriminant analysis (LDA) (36) to recognize each of the gestures performed. LDA is a commonly used pattern recognition algorithm for prosthetic control (37) and already commercialized by COAPT LLC (Chicago, USA) (38, 39). We chose it for the ease of implementation, classification speed, and high accuracy compared to other similar approaches (40). The 10 steady-state segments for every gesture were concatenated and created a 10-s vector. We trained the classifier by extracting four time-domain features from the raw segmented data including mean absolute value, zero crossing, slope sign change, and waveform length (41). We chose for a feature extraction window of 200 ms (with an overlap of 100 ms), which would be within acceptable range for real-time myoelectric applications (42). The classifier was validated with a three-split Monte Carlo cross-validation approach (43). Each time, a different part of the segmented data was used for training (always 70%) and testing (always 30%). The average off-line classification accuracy of these three trainings was used as performance metric per participant. Additionally, we tested how the offline classification accuracy per participant was affected by the number of gestures that had to be classified.

## Results

### Motor Control Properties

#### Activation Pattern Repeatability

As illustrated in Figure 4, DMD individuals exhibited comparable correlation values to healthy individuals. The average *R*^2^ coefficient was 0.89 ± 0.12 (mean ± SD) for DMD and 0.89 ± 0.13 for healthy participants between repetitions. An example of the 10 repetitions for a DMD participant can be seen in Figure 2.

#### Spatiotemporal Activation Patterns

Figure 5A shows the normalized and absolute activations of both participant groups. The normalized activation was on average higher for the DMD (0.63 ± 0.23) than for the healthy participants (0.26 ± 0.2). The maximum normalized value observed for participants with DMD was one (only DP3) and the minimum 0.3 (DP2), while for healthy were, respectively, one (only HP1) and 0.05 (HP8). The maximum absolute activation of the DMD participants was on average 35 ± 19 μV, while for healthy individuals, it was 89 ± 358 μV. The maximum value observed for participants with DMD was 108 μV (DP1) and the minimum 18.6 μV (DP3), while for healthy, were. Respectively. 628 μV (HP1) and 8.5 μV (HP8). Due to the difference in the number between the healthy and DMD participants, we have fewer repetitions for the DMD individuals, i.e., seven gestures multiplied by 10 repetitions per participant, which means 210 for the DMD vs. 560 for the healthy. Figure 5B shows the COG for the seven gestures in the electrode space for both participant groups. Healthy participants show a broader spatial distribution for the seven gestures. Wrist flexion and close hand appear to be spatially close. Along the dorsoventral direction, on average, thumb extension was at the dorsal limit (COGx = 18.9%), while close hand was at the ventral limit (COGx = 68%). In the proximodistal direction, wrist flexion was at the proximal limit (COGy = 41%), and thumb flexion was at the distal limit (COGy = 75%). Participants with DMD showed on average a close clustering of the seven gestures. Thumb extension and wrist flexion were the most spatially close gestures. In the dorsoventral direction, on average, open hand was at the dorsal limit (COGx = 20%), while close hand was at the ventral limit (COGx = 61%). In the proximodistal direction, the same gestures were again the limits, with close hand being the proximal (COGy = 47%) and open hand the distal (COGy = 69%).

#### Activation Pattern Dimensionality

The participants with DMD needed on average three PCs to explain >90% of the total variance of the seven gestures and 10 repetitions (Figure 6A). The same variance threshold was crossed on average by five PCs for the healthy participants. For the healthy group, PC1 explained 45%, while the same component explains 61% of the total variance for the DMD group.

Figure 6B shows which gestures were the most similar (by means of the squared Pearson correlation). The healthy participants exhibited correlations *R*^2^ > 0.3 on average between two gestures. The highest correlations were found between hand open and thumb extension (0.39 ± 0.2) and wrist extension (0.36 ± 0.17). The participants with DMD exhibited *R*^2^ > 0.3 on average across nine gestures. Those were found between close hand and wrist flexion (0.45 ± 0.23); between hand open and index extension (0.38 ± 0.23), thumb extension (0.46 ± 0.33), thumb flexion (0.34 ± 0.27), and wrist extension (0.62 ± 0.23); between index extension and thumb extension (0.47 ± 0.15) and thumb flexion (0.36 ± 0.04) and wrist extension (0.31 ± 0.16); and finally between thumb extension and thumb flexion (0.42 ± 0.21).

### Gesture Recognition for Myocontrol

**Figure 7:**
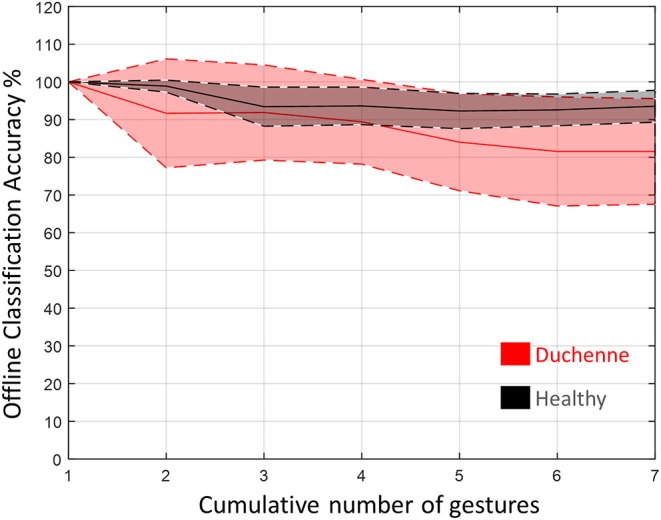
The difference in average off-line classification accuracy for healthy and Duchenne muscular dystrophy (DMD) participants as a function of the gestures needed to be identified by the linear discriminant analysis (LDA) classifier. The full lines represent the mean and the dashed the standard deviation.

The LDA classifier was trained using the seven gestures. Figure 7 shows the results of the off-line classification accuracy as a function of the gestures that had to be recognized. The average off-line classification accuracy of the DMD participants was always lower than the average of the healthy participants. When all the gestures were included, this accuracy reached 93.6 ± 4.2% for the healthy and 81.6 ± 14% for the DMD participants. The off-line accuracy stopped dropping at six gestures for the participants with DMD, while for the healthy participants, this happened at three (Figure 7).

## Discussion

In this study, we measured HD-sEMG activity from the forearm of eight healthy and three DMD participants during seven hand/wrist-related tasks. We performed analyses in order to characterize the differences in activation patterns shape, repeatability, and dimensionality, as well as gesture recognition between healthy and DMD individuals.

The three participants with DMD showed motor control alterations in terms of dimensionality and spatiotemporal activations compared to the healthy population, supporting our hypothesis. These alterations were mainly expressed by the COGs across DMD gestures being more closely located than COGs across healthy gestures and comfortable muscle activations close to their maximal contraction level (0.63 ± 0.23). Also, participants with DMD showed a higher correlation between gestures, and when their gesture space was decomposed to its PCs, 90% of it was explained by fewer components (3) than for the healthy (5). Differences were also found between DMD participants likely due to different stages of the disease. However, in terms of repeatability per gesture, the two populations showed an unexpected clear similarity. Despite the consequences of muscular degeneration and minimal hand/wrist motion (especially for DP3, Supplementary Video), the myocontrol potential for the DMD participants is remarkably present and comparable to the healthy participants, disproving our hypothesis. However, the existing differences, due to the specificities of individuals with DMD, need to be addressed while developing myocontrol algorithms.

The results indicated that repeatability was intact for the participants with DMD and comparable to that of the healthy participants (Figure 4). This is an important requirement for robust and repeatable pattern recognition-based myocontrol (44) of assistive robotics.

Participants with DMD exhibited lower absolute activations and higher normalized activations compared to the healthy participants (Figure 5). This shows that participants with DMD operate closer to their maximum effort, as opposed to healthy participants, in order to perform simple hand/wrist-related tasks, and yet they produce lower absolute sEMG activity. This constant high effort can have detrimental consequences for the muscle integrity of people with DMD and even speed up disease progression or lead to disuse of the hand. Assistive wearable robotics may be able to decrease the mechanical load on the muscles and promote daily use (7, 45). This result, together with the fact that the most progressed participant (DP3) presented simultaneously the maximum normalized and the minimum absolute sEMG activity, agrees with previous studies stating that the disease progression results in lower absolute sEMG amplitude (46) and also in higher effort and fatigue (47). DP1 and DP3 exhibited comparable trends between each other. For DP1, we observed high absolute activations (around 100 μV, comparable to healthy participants) and on average medium absolute activations. DP2 showed lower absolute activations (around 60 μV), however also medium absolute activations. Regarding spatial distribution of the activation patterns, healthy participants showed lower spatial similarity than participants with DMD (Figure 5B). It appears that on average for the DMD participants, the seven gestures used in this study, engaged only a subset of the electrodes, closely clustered to each other compared to the healthy participants. Similarly, to lower spatial similarity, healthy participants (Figure 6) exhibited a higher degree of dimensionality, as expressed by the larger repertoire of orthogonal and uncorrelated sEMG patterns they can produce across the seven hand/wrist-related gestures and 10 repetitions. The healthy population needed five PCs to explain at least 90% of the variance in the original data, while DMD participants needed three, except DP1 that needed four. Additionally, the higher correlation between the gestures points toward the fact that, in terms of sEMG activation patterns, there is more similarity in DMD. This may provide another indication (together with variability in maximum activation) of how the progress of the disease affects motor control, since DP1 is the least affected participant. The decrease in dimensionality may be partially attributed to the increased level of co-contractions between agonist and/or antagonist muscle groups that we observed in the DMD participants when performing the tasks and further supported by a recent hand motor performance study in people with DMD (48). Co-contractions may be elicited by the effort of the participants to stabilize their wrist during the tasks, but further work is needed to explore this hypothesis.

According to the muscle synergies hypothesis, the CNS uses specific simplified commands (muscle synergies) in order to act efficiently upon the redundant musculoskeletal system and complete a motor task (49). In the case of DMD, the intact CNS and neural pathways are acting upon a progressively limited musculoskeletal system. This may lead to progressive adaptations in the CNS, similar to those observed in stroke survivors (50) expressed via compensatory movements (51), co-contractions, and lower dimensionality. Regarding gait analysis in DMD, it was shown that gait motor control complexity is minimally affected by the disease (in the early stages) (52); however, for the more complex hand and wrist control, there is no evidence. Considering the sEMG measurement for the participants with DMD as the neural output (53), it is not yet understood if the observed commonalities between different gestures can be attributed to the impaired musculoskeletal system (i.e., more similarities in how motor units process incoming axonal spike trains) or to adaptations in the CNS (i.e., increased common synaptic input to alpha motor neuron pools). Future work will employ HD-sEMG in combination with decomposition techniques (54) in people with DMD to provide further insights.

According to our results, there is potential for the robust decoding of hand/wrist motor intention in individuals with DMD. This can enable individuals with DMD to control a high-tech hand orthosis with multiple degrees of freedom. However, there was a noticeable decay of the LDA off-line classification performance when more gestures were added for the participants with DMD, which was larger than the one for the healthy participants (Figure 7). Despite the lower performance, the classification performance is on average larger than 80% for all the seven gestures and more or equal to 90% for up to four gestures. Together with the ability of the DMD participants to create repeatable HD-sEMG activation patterns, this result shows the potential of myocontrol for decoding of hand/wrist motor intention across a key selection of gestures. Currently, the implementation of HD-sEMG in dynamic control of wearable exoskeletons is limited by a number of factors, such as the number of cables between amplifiers and electrodes, as well as large amplifiers. This lack of portability restricts measurements in dynamic conditions (motion) and induces movement restrictions, user discomfort, and signal artifacts (23), therefore limiting control of wearables. However, recent developments show promising steps toward more portable amplifiers that reject movement artifacts and powerline interference, while at the same time do not obstruct movements and ensure tight placement of the electrodes (23, 55). To this point, the main limitation of portable amplifiers is the relatively limited number of electrodes provided [32 (23) and 16 (55)]; however, they open new avenues for HD-sEMG control of exoskeletons.

The current performance of classification could be optimized with the development of DMD-tailored classification algorithms, which will take into account the specificities of the disease. Such specificities are the progression of the disease (co-contractions and fatigue), the low sEMG signal to noise ratio (46), and the differences in the motor control strategies (higher spatial similarity, lower dimensionality in terms of orthogonal and uncorrelated sEMG patterns, higher activation levels during low-intensity tasks). Further tailoring can be made by building numerical neuromusculoskeletal models of specific DMD individuals that can provide additional features for classification (56–58). The observed lower spatial dimensionality in the HD-sEMG may suggest that sEMG data compression before classification might be a strategy due to the lower variability that individuals with DMD present. This can be achieved by first lowering the dimensionality of the feature space of the raw data based on dimensionality reduction techniques such as PCA or partial least squares (PLS) (59) and use the resulting data as an input to an LDA classifier. Further, reduction of the number of electrodes can be achieved based on detection of heatmap areas carrying common and individual information (19). The higher spatial similarity is an important finding of this study that can be considered for guiding such decisions. More extensive research with individuals with DMD is necessary to identify the relevant feature space and test the performance of various classifiers and electrode numbers and configuration in order to inspire DMD customized classifiers.

We included in our case study three participants with DMD with large functional variability in order to explore a larger spectrum of the disease instead of a cluster of cases with similar characteristics. However, DMD is a disease with large functional heterogeneity due to different progression patterns (60) and our limited sample does not cover the complete spectrum. However, our study is limited by the low number of participants with DMD. This is an unavoidable limitation due to the low number of available participants. We also intended to comply with the ethical and legal standards while conducting our study by not recruiting participants who are already involved in other studies at the same time. Hence, our conclusions and results need to be taken as indicative until research is performed with more participants, which will allow for more general and strong conclusions.

Additionally, we did not monitor the level of contraction during the conduction of the measurements. We explicitly asked our participants to perform all movements comfortably, but we did not control this condition. It is known from the literature that different contraction levels elicit a small shift in the main activity area, however not significantly altering the spatial distribution of HD-sEMG in the forearm (14). Another limitation of this study is that the selection of the seven gestures used for acquiring and analyzing the data was based on gestures involved in common ADL (25), and each gesture was analyzed separately. However, in reality, ADL involves multiple combinations of the selected gestures in some case simultaneously, which would result in the activation of more than one muscle region when combined finger and wrist movements are occurring in order to allow for object grasping and manipulation. In such case, the spatial distribution of the sEMG activations will not be so clearly segmented. Therefore, before applying our findings for myocontrol targeting ADL, we need to take caution and further test the validity of our findings in situations demanding a higher degree of complexity (combination of gestures).

Future work will evaluate the application of our protocol to more participants with DMD in order to investigate further the characterization of forearm electromyograms for individuals with DMD and come to more general conclusions regarding this very diverse population. Moreover, we are interested in the exploration of online classification performance implemented outside of the lab in order to resemble daily-use conditions. An extended protocol in order to decode the neural drive (54) in DMD would offer further insights regarding the source of the differences in hand/wrist motor control observed in our analysis between participants with DMD and healthy participants. Next to that, the use of non-negative matrix factorization (NMF) may give further insights regarding muscle group synergies in hand movements in DMD (61). Lastly, an analysis of the homogeneity of activations needs to be carried out using HD-sEMG, as it is known that different joint positions and contraction strength and duration may cause muscles to activate in a non-homogeneous manner (21). The results of this study together with the future studies will be further used for the development of myocontrol algorithms for the robust control of an active hand exoskeleton (29, 62), developed within the Symbionics project (63) for individuals with DMD.

## Conclusion

We characterized the forearm electromyograms spatiotemporally of three individuals with DMD and compared to eight healthy individuals. For the first time, we propose a systematic analysis on how the disease affects the distribution of HD-sEMG pattern in the forearm and the repeatability and activation distribution of these patterns. Additionally, we explored the potential for the myocontrol via decoding of motor intention from the forearm muscles of individuals with DMD. We performed this study in order to get a better understanding of DMD hand/wrist motor control with regard to exoskeleton applications. Future studies will focus on testing sEMG for the real-time decoding of hand/wrist motor intention with individuals with DMD. Moreover, we will implement and test the feasibility of sEMG control with a new active hand exoskeleton for individuals with DMD.

## Data Availability Statement

The datasets generated for this study can be found in the 4TU repository [https://data.4tu.nl/]. DOI: https://doi.org/10.4121/uuid:f252f933-90be-4543-9c13-3c4efe208052.

## Ethics Statement

The studies involving human participants were reviewed and approved by Medical Ethics Committee (METC) of Twente, protocol number: NL59061.044.16. The patients/participants provided their written informed consent to participate in this study.

## Author Contributions

KN performed the main review of literature and research protocol development, data acquisition and analysis, creation of figures, and drafting of the manuscript. NR participated in the data acquisition, research protocol development, creation of figures, and made critical revision of the manuscript. RM participated in the measurements and partially in data analysis and proofread the document. JN participated in the PCA and performed the off-line LDA classification. MS oversaw the complete writing process and design of data analysis procedures. BK was actively involved in the writing process and made substantial revisions of the manuscript. All authors read and approved the final manuscript.

### Conflict of Interest

The authors declare that the research was conducted in the absence of any commercial or financial relationships that could be construed as a potential conflict of interest.

## Supplementary Material

Video S1 Characterization of muscle activation diversity in Duchenne muscular dystrophy via high-density electromyography: implications for myoelectric control.
